# Energy-Efficient Cognitive Radio Sensor Networks: Parametric and Convex Transformations

**DOI:** 10.3390/s130811032

**Published:** 2013-08-21

**Authors:** Muhammad Naeem, Kandasamy Illanko, Ashok Karmokar, Alagan Anpalagan, Muhammad Jaseemuddin

**Affiliations:** 1 ELCE Department, Ryerson University, 350- Victoria Street, Toronto, ON M5B 2K3, Canada; E-Mails: muhammadnaeem@gmail.com (M.N.); killanko@ryerson.ca (K.I.); ashok@ryerson.ca (A.A.); jaseem@ee.ryerson.ca (M.J.); 2 Department of Electrical Engineering, COMSATS Institute of IT, Wah Campus, Wah, Pakistan

**Keywords:** power allocation, nonlinear optimization, cognitive radio sensor network

## Abstract

Designing energy-efficient cognitive radio sensor networks is important to intelligently use battery energy and to maximize the sensor network life. In this paper, the problem of determining the power allocation that maximizes the energy-efficiency of cognitive radio-based wireless sensor networks is formed as a constrained optimization problem, where the objective function is the ratio of network throughput and the network power. The proposed constrained optimization problem belongs to a class of nonlinear fractional programming problems. Charnes-Cooper Transformation is used to transform the nonlinear fractional problem into an equivalent concave optimization problem. The structure of the power allocation policy for the transformed concave problem is found to be of a water-filling type. The problem is also transformed into a parametric form for which a *ε*-optimal iterative solution exists. The convergence of the iterative algorithms is proven, and numerical solutions are presented. The iterative solutions are compared with the optimal solution obtained from the transformed concave problem, and the effects of different system parameters (interference threshold level, the number of primary users and secondary sensor nodes) on the performance of the proposed algorithms are investigated.

## 1. Introduction

The energy-efficiency of wireless sensor devices and the scarcity of wireless bands are two major design parameters in any wireless sensor network (WSN), due to limited battery power of these devices and the fixed natural wireless spectrum. Intelligent and optimal use of electrical energy is also of paramount importance in order to reduce green house gas emissions. Recent studies show that wireless communication and sensor networks will be responsible for a significant portion of the total green house gas emissions, due to their predicted exponential growth in the near future [1]. Therefore, energy-efficient design is indispensable for future WSNs. The problem of wireless bands is even more exacerbated, because of the static spectrum allocation policy. As a result, some spectra are heavily used, and some, on the other hand, are heavily under-utilized in time, frequency or space. This fact has given birth to opportunistic spectrum access techniques in cognitive radio networks, where the cognitive user can sense and access the licensed spectrum dynamically while it is not in use. However, the secondary sensor nodes (SSNs) need to make sure that at any time, they do not exceed the total interference limit that they inherently generate on the primary users.

Cognitive radio is seen as an effective approach for higher spectral and energy efficiency in wireless communication systems for two reasons. Firstly, the energy efficiency related functionalities can be embedded into the cognitive operational cycle. Secondly, from the green perspective, the spectrum is a natural resource, which should not be wasted on idle licensed channels, but be shared efficiently [2].

Wireless sensors play a very important role in many applications, such as industrial monitoring [3], environmental (air/water quality) monitoring [4], health monitoring [5], seismic vibration sensing, *etc*. They have found applications in many *ad hoc*, military and commercial wireless systems. Due to the rapid growth of WSNs in every facet of our daily lives, the limited frequency spectrum available for WSN applications will be heavily crowded. Therefore, WSNs using cognitive radio techniques are envisioned for future WSNs [6].

Since the sensor devices are powered by battery and often are embedded into the system permanently, it is often impractical to charge or replace the exhausted battery. Therefore, energy-efficiency is most important for sensor operations and communication over the wireless channels. While energy efficiency is the most important parameter in designing sensor networks, other quality of service (QoS) parameters, such as throughput and delay, need to be maintained. An energy efficiency metric can be defined as the effective throughput per one unit of transmitted power. That means, we can call a technique energy-efficient or green if we can reduce the total network power without introducing significant impact on the network throughput. Energy efficiency is measured in bits per Joule. This means energy is required in Joules to transfer one bit from one point to the other. The term green is synonymous to energy-efficiency for WSN design, since maximizing energy efficiency reduces the power usage in a WSN lifecycle and, consequently reduces green house gas emissions. In this paper, we investigate the optimal power allocation in the uplink cognitive wireless sensor network, where each sensor node is transmitting its sensed data to the centralized fusion center (either the cluster head or a secondary base station). Our objective is to maximize the energy efficiency under the primary user interference constraint, so that the sensed data is transmitted with the minimum possible power, and as a result, the battery life of the sensor (and, hence, the sensor network life) is maximized.

### 1.1. Related Work

Below, we discuss some literature on WSNs that deals with the cognitive techniques to minimize energy and/or other related issues. The authors in [7] studied minimization of the energy consumption per bit for a distributed cognitive radio sensor network. The energy efficiency (EE) in joint source and channel sensing for cognitive radio sensor networks is considered in [8]. In [9], the optimal power allocation of a sensor node in a cognitive radio environment is analyzed, and the optimum solution is derived for fading channels. Residual energy-aware channel assignment in a cluster-based multi-channel cognitive radio sensor network is studied in [10]. In [11], the design and implementation issues for a frequency-agile WSN are analyzed. Event detection and channel allocation in two-tier cognitive radio sensor networks using a constrained Markov decision process is discussed in [12]. In [13], the authors discussed the advantages of a holistic approach to cognition in wireless sensor networks. This is achieved by incorporating learning and reasoning in the upper layers and opportunistic spectrum access at the physical layer.

Energy-efficient spectrum sensing for cognitive wireless sensor networks is studied in [14]. The distributed sensing technique optimizes the energy efficiency with constraints on the minimum target probability of detection and the maximum permissible probability of false alarms by choosing the sleeping and censoring design parameters. Although the energy efficiency issue is considered for the sensing spectrum, it is not considered for transmitting the information data to the centralized receiver. A number of studies used game theory to optimize energy efficiency [15–17]. The game theory approach to energy efficiency does not maximize the total system energy efficiency. Game theory finds a competitive equilibrium among the users who try to maximize their individual energy efficiencies simultaneously. In the above papers that use game theory, the channel model is not OFDMA; rather, it is CDMA. In these channels, a users' selection of power level will affect the EE of another user. Because of this, game theory is ideal for these channels. In our work, the channel model is OFDMA, where the channels are orthogonal. One user's energy level does not affect the other users’ throughput or energy efficiency.

The tradeoff between energy and spectral efficiency in OFDMA networks is investigated in [18]. After showing that EE is quasi-concave in spectral efficiency (SE), the authors use an approximation on this relation to obtain an algorithm that is used for power and subcarrier allocation. EE in the uplink of an OFDMA system is addressed in [19], where users’ average EE is defined as the ratio of moving averages of the data rate and power expenditure. The instantaneous transmission rate and power allocation that approximately maximizes the average EE is obtained through a low-complexity solution. A delay-aware data collection network structure for WSNs is proposed in [20], where the objective is to minimize delays in the data collection processes of WSNs. Two network formation algorithms are designed to construct the network structure in both the centralized and decentralized schemes. Their work assumed that the sensors are communicating on the dedicated channels. Cross-layer optimization of fusion, power allocation and delay for a cognitive sensor network is given in [21], where the goal is to minimize the mean distortion of the system under constraints on the total energy consumption and the total delay. The work, however, did not consider any spectrum access and interference issues for the primary user.

Authors in [22] focus on the joint optimization of the medium access and the physical layers of a cognitive radio network. This paper finds the optimal transmission duration and power allocation that maximizes EE. The energy consumption of an OFDMA cognitive radio network is studied in [23]. The EE of the network is maximized by the use of an algorithm that jointly optimizes the modulation and the power allocation. In [24], opportunistic power allocation and sensor selection schemes for WSNs are analyzed in the context of the minimization of power, distortion and the enhancement of network lifetime.

### 1.2. Contributions

Although energy-efficient techniques for WSNs exist in the literature, according to the best knowledge of the authors, there is no power allocation scheme that deals with the analysis and optimization of the EE metric in a cognitive radio-based WSN. The motivation of this work is to fill the gap, especially important for future green radio communication, with the aim of analyzing the power allocation problem that maximizes the EE (bits/Joule/Hz).

The main contributions of this paper are summarized as follows:

1. We propose a constrained optimization problem that maximizes the EE for an uplink wireless sensor network utilizing cognitive radio techniques. First, we show that the proposed optimization problem is a fractional programming problem. We use Charnes-Cooper transformation to transform the formulated concave fractional program (CFP) into an equivalent concave optimization problem that has a water-filling type power allocation rule.
2. Then, we present an iterative *ε*-optimal solution for the CFP. The proposed algorithms are based on the Dinkelbach method for CFP. We show that it converges to the *ε*-optimal solution point. The proposed *ε*-optimal algorithm provides a practical solution for power allocation in energy-efficient cognitive radio networks.
3. We present performance analysis of a *ε*-optimal algorithm with the simulation results.

### 1.3. Notations and Organization

Unless otherwise specified, we use ***A***, ***a*** and *a* to represent matrix, vector and an element of a vector, respectively. When *a_i_* ≥ 0 for all components, *i*, of a vector, ***a***, we use ***a*** ≥ 0. We use the following expression, (*a*)^+^ ≜ max(0, *a*), to ensure the positivity of a real number. We use log_2_ as log and log*_e_* as ln. The most used notations and symbols are presented in Table 1.

The paper is organized as follows. The system model and problem formulation are presented in Section 2. In Section 3, we present the optimal power allocation scheme using concave fractional programming. The *ε*-optimal algorithms and their convergence are presented in Section 4. We present simulation results in Section 5 and conclude in Section 6.

## 2. System Model and Problem Formulation

**Figure 1.**
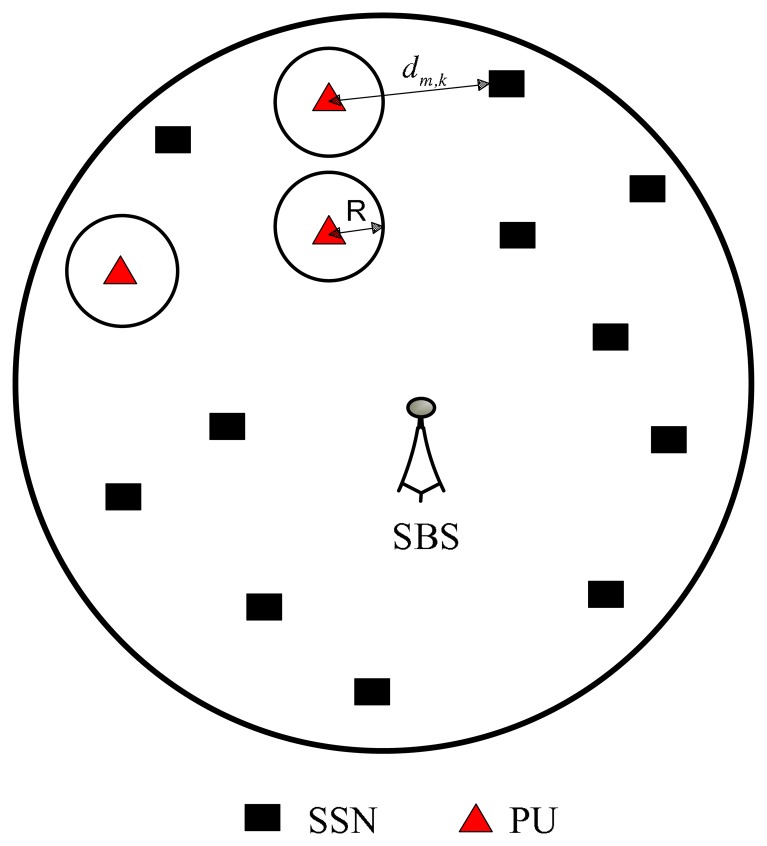
A cognitive radio system model with a secondary base station (SBS), secondary sensor nodes (SSNs) and primary users (PUs).

We consider a cognitive radio-based wireless sensor network, where a set of sensor nodes are communicating with a centralized secondary base station (SBS), as shown in Figure 1. Since the sensor nodes are communicating using a channel licensed to the primary users (PUs), we call these sensor nodes secondary sensor nodes (SSNs). We assume that each active SSN communicates on an orthogonal frequency band, so that it will not introduce interference to the other SSNs. Suppose that the number of SSNs and PUs are *K* and *M*, respectively. Note that *M* PUs can mean either *M* PU devices or *M* geographic locations or regions in which the strength of the SSN signals must be constrained. Transmissions to each SSN take place on a separate, preassigned subchannel, and a central controller decides the channel assignment and power level. We denote by *p_c_* the static circuit power of the source in the transmit mode [18], *p_k_*, the source transmitted power of the *k*th SSN, *I_m_*, the interference threshold at the *m*th PU and *h_k_*, the channel from the *k*th source SSN to the SBS.

Two schemes for PU protection are recommended in the IEEE802.22 WRANstandard for cognitive radio networks. They are listen-before-talk using spectrum sensing techniques and geolocation using the available database [25]. The SSN senses the presence of primary network signals in the first scheme in order to select the channels that are not in use. On the other hand, in the geolocation/database scheme, the locations of primary and secondary sensor nodes are stored in a central database. The central controller (SBS) of the SSNs has access to the location database. In this paper, we assume the latter scheme, that is, the SBS gets the location information of each PU from the central database. We also assume that SBS has knowledge of PUs’ channel gains.

As shown in Figure 1, there is a PU protection area, wherein the strengths of the SSN signals must be constrained. We define as *R*, the radius of the protected circular area for each individual PU. Given a distance, *d_m,k_* between the *k*th SSN transmitter and the *m*th PU and the radius, *R_m_*, of the protected circular area of the *m*th PU, the channel from the source to the *m*th PU in the *k*th SSN frequency band is given as:

(1) $$g_{m,k}={\tilde{g}}_{m,k}G_{o}{\left(\frac{d_{o}}{d_{m,k}-R_{m}}\right)}^{\beta },$$

where *g̃_m,k_* is the small scale fading, *d_o_* is the reference distance for the antenna far field, *G_o_* is the constant and *β* is the path loss exponent [26]. Without loss of generality in sequel, we assume that *R*_1_ = *R*_2_ = ⋯ =*R_M_* = *R*.

In this problem, our goal is to maximize the energy efficiency of the SSN's transmissions and at the same time, meeting the interference constraints imposed by the PUs. The energy efficiency (EE) metric that we use in this paper is defined as throughput (bits/sec/Hz) per unit transmission power (in Watts) [27]. For our system, we can express EE in information bits per Joule as follows:

(2) $$\Gamma \left(p\right)=\frac{\sum_{k=1}^{K}C_{k}}{p_{c}+ϒ\sum_{k=1}^{K}p_{k}}$$

where ϒ is the power amplifier scaling factor [28,30] and $C_{k}=\log\left(1+\frac{p_{k}h_{k}}{N_{0}}\right)$ is the maximum theoretical spectral efficiency (SE) (bits/s/Hz), according to the Shannon capacity formula on the *k*th SSN link to the SBS. Mathematically, we can write the EE maximization problem for SSNs as:

(3) $$\begin{array}{cc} \underset{p}{\text{maximize}} & \;\Gamma \left(p\right)\\ \text{subject to} & \\ & C1:\sum_{k=1}^{K}p_{k}g_{m,k}\leq I_{m},\forall m\\ & C2:p_{k}\geq 0,\forall k \end{array}$$

In Equation (3), the constraint, *C*1, assures that interference to primary users is less than a specified threshold.

**Figure 2.**
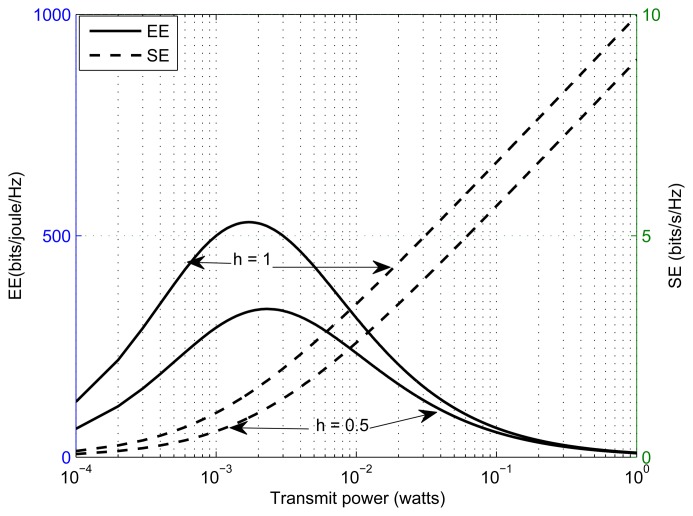
Plots for energy efficiency (EE) and spectral efficiency (SE) as a function of transmitted power.

Typical variation of EE and SE with transmitted power for the single user case is shown in Figure 2 for two scenarios of the channel gains. In the first scenario, channel gain between the SSN and SBS is set to 0.5, and in the second scenario, channel gain between the SSN and SBS is set to one. From Figure 2, we can observe that EE achieves a maximum, while SE continues to increase with the transmitted power. We can say that the optimum SE solution does not mean the optimum EE solution. This is because EE is not an increasing function of the transmitted power, while spectral efficiency is a non-decreasing function of the transmitted power.

## 3. Optimal Power Allocation as Concave Fractional Programming (CFP)

In this section, we first introduce the concave fractional program (CFP) and, then, show how we can convert our problem into an equivalent concave program.

**Fractional Program (FP):** An optimization problem is called a fractional program when the objective function is the ratio of two functions. A FP problem is expressed as:

(4) $$\begin{array}{cc} \underset{x\in X}{\text{maximize}} & \;\frac{f\left(x\right)}{g\left(x\right)}\\ \text{subject to} & \\ & h_{i}\left(x\right)\leq 0,\forall i=1,2,\cdots ,N \end{array}$$

where *f*(), *g*() and *h_i_*(), *i* = 1, 2, ⋯, *N* denote real-valued functions, which are defined on the set, *X* of ℝ*^n^* [31].

**Concave Fractional Program (CFP):** The FP in Equation (4) is a concave fractional program if it satisfies the following two conditions: (i) *f() is concave and g() is convex on X*, (ii) *f() is positive on S if g() is not affine*, where *S* = {*x* ∈ *X : h_i_*(*x*) ≤ 0, ∀i = 1, 2, ⋯, *N*} [31].

It can be noted that, in a CFP, any local maximum is a global maximum, and in a differentiable CFP, a solution of the KarushKuhnTucker (KKT) conditions provide the maximum [32,33]. It is seen from Equation (3) that the function in the numerator is a concave function and that the denominator is affine, and all the constraints are affine. We can see that the optimization problem, Equation (3), is differentiable and satisfies the conditions of CFP. Therefore, any local maximum is a global maximum, and the KKT conditions give the optimal solution of Equation (3).

A CFP with an affine denominator can be reduced to a concave program with Charnes-Cooper Transformation (CCT)[32,34]. Therefore, using CCT, the equivalent concave program of problem (3) can be written into the following form:

(5) $$\begin{array}{cc} \underset{\frac{y}{t}\in X,t>0}{\text{maximize}} & \;tf\left(\frac{y}{t}\right)\\ \text{subject to} & \\ & th_{i}\left(\frac{y}{t}\right)\leq 0,\forall i=1,2,\cdots ,N\\ & tg\left(\frac{y}{t}\right)=1 \end{array}$$

Problem (4) has an optimal solution, if and only if Equation (5) has an optimal solution. The two solutions are related to each other with the relation, *y* = *tx* and $t=\frac{1}{g\left(x\right)}$. Now, we focus on our optimization problem, Equation (3). With Charnes-Cooper transformation (CCT), we can write an equivalent concave program for our optimization problem, Equation (3), as:

(6) $$\begin{array}{cc} \underset{y,t>0}{\text{maximize}} & \;t\sum_{k=1}^{K}\log\left(1+\frac{y_{k}h_{k}}{tN_{0}}\right)\\ \text{subject to} & \\ & C1:\sum_{k=1}^{K}y_{k}g_{m,k}-tI_{m}\leq 0,\forall m\\ & C2:t_{pc}+ϒ\sum_{k=1}^{K}y_{k}=1\\ & C3:y_{k}\geq 0,\forall k \end{array}$$

The following theorem is obtained from the KKT conditions and gives the structure of the allocated powers of the SSN transmitter.

**Theorem 1.** *The power profile for which the total energy efficiency is maximized for* Equation (6) *is*:

(7) $$p_{k}^{*}=\left(\frac{y_{k}^{*}}{t^{*}}\right)={\left(\frac{1}{\eta_{k}}-\frac{1}{\xi_{k}}\right)}^{+},\forall k$$

*where* $\eta_{k}=\ln2\left(v+\sum_{m=1}^{M}\phi_{m}g_{m,k}\right)$, *Φ_m_ and υ are Lagrange multipliers that are yet to be determined and* $\xi_{k}=\frac{h_{k}}{N_{0}}$, $y_{k}^{*}=t^{*}{\left(\frac{1}{\eta_{k}}-\frac{1}{\xi_{k}}\right)}^{+}$ *and* $t^{*}=\frac{1}{p_{c}+ϒ{\sum_{k=1}^{K}\left(\frac{1}{\eta_{k}}-\frac{1}{\xi_{k}}\right)}^{+\cdot }}$.

*Proof:* In order to keep the continuity of our discussion, the proof of Theorem 1 is given in the Appendix A.

We can see that the solution of the above problem is similar to the water-filling algorithm. However, Equation (7) needs to determine (*M*+1) Lagrangian multipliers. We call this water filling energy-efficient water-filling in cognitive radio networks. Since Equation (6) is a concave optimization problem, we can determine the Lagrangian multipliers with dual optimization. We can write the Lagrangian dual objective function as:

(8) $$D\left(\phi ,\upsilon \right)=\text{mazimize}\;L\left(y^{*},t^{*},\phi ,\upsilon \right)$$

and the dual optimization problem is:

(9) $$\begin{array}{cc} \underset{\phi \geq 0,\upsilon }{\text{minimize}} & \;D\left(\phi ,\upsilon \right) \end{array}$$

The dual function needs to be minimized over *ϕ* and *υ* to obtain the optimal dual solutions, ϕ* and υ*. The problem, Equation (9), can be solved with any gradient algorithm [33].

We illustrate the water-filling scheme with two example scenarios. We consider a cognitive radio network with six SSNs and one PU. We set the interference threshold to 10^−6^*μ*W. We consider two scenarios with different channel conditions that are given below.

1. *h_k_* = (0.3 0.2 0.1 0.09 0.05 0.03)
  *g_m,k_* = (0.001 0.002 0.003 0.004 0.005 0.006)
2. *h_k_* = (0.6 0.4 0.3 0.09 0.08 0.1)
  *g_m,k_* = (0.006 0.0001 0.00003 0.0041 0.0005 0.00002)

**Figure 3.**
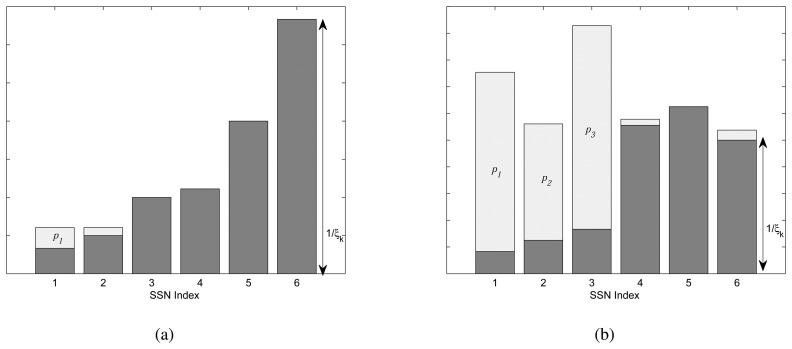
Two example scenarios showing the water-filling allocation for different channel gain and interference gain ratios. (**a**) Scenario 1: When SSN with a good channel to SBS has less interference to the PU. (**b**) Scenario 2: When SSN with a good channel to SBS has more interference to the PU.

Note that *h_k_* and *g_m,k_* are, respectively, channel gains from *k*th SSN to SBS and from the *k*th SSN to the *m*th PU. In the first scenario, SSNs with a good channel to SBS generate less interference to the primary network. In the second scenario, SSNs with a good channel to SBS generate more interference to the primary network. In Figure 3, $\frac{1}{\xi_{k}}\;\left(\text{where}\;\xi_{k}=\frac{h_{k}}{N_{0}}\right)$ for each SSN is plotted along with its allocated power level. From the figure, we can observe that even if an SSN has a very good channel with the SBS, if it is causing more interference to the PU, then it gets less power. In Figure 3a, only SSN-1 and SSN-2 have allocated power, because of (i) a good channel between them and SBS and (ii) less interference to the PU. If there is a substantial difference in the channel gains, then SSNs that cause less interference to the PU get more power. This is illustrated in Figure 3b, where the channel gain between SSN-3 and SBS is less than SSN-1 and SSN-2; however, SSN-3 gets more power allocation, because it has a very weak channel link with the PU.

## 4. Iterative Algorithm

In this section, we present an iterative algorithm to solve the optimization problem given in Equation (3). We show that the proposed algorithm is a *ε*-optimal algorithm (for any *ε* > 0, *ε*-optimal algorithms guarantee the solution within *ε* of optimal). This algorithm is based on the Dinkelbach method for fractional programming problems [35]. In the Dinkelbach approach, the fractional objective is transformed into a parametric optimization problem. Consider the following general optimization problem, where *x* ∈ ℝ^*n*^ and *q* ∈ ℝ.

(10) $$\begin{array}{cc} \underset{x}{\text{maximize}} & \frac{N\left(x\right)}{D\left(x\right)}\\ \text{subject to} & x\in S \end{array}$$

The parametric problem associated with Equation (10) can be written as:

(11) $$\begin{array}{ll} \underset{x}{\text{maximize}} & N\left(x\right)-qD\left(x\right)\\ \text{subject to} & x\in S \end{array}$$

The equivalence between the fractional programming problem (10) and the parametric programming problem (11) is discussed in [35]. The Dinkelbach theorem for the equivalence can be written as follows:

**Dinkelbach Theorem:** q* = *N*(x*)/*D*(x*) = max{*N*(*x*)/*D*(*x*)|*x* ∈ *S*}, if and only if max{*N*(*x*) − q*D(*x*)|*x* ∈ *S*} = *N*(x*) − q*D(x*) = 0.



**Algorithm 1** Iterative algorithm.
 **Initialization:** *q* ← 0 *ε* ← 10^−6^ *i* ← 0 *Convergence* ← *false*, **Define:**
$\gamma \left(p,q\right)\leftarrow \sum_{k=1}^{K}C_{k}-q(p_{c}+ϒ\sum_{k=1}^{K}p_{k})$ **while** (*Convergence* = *false*) *and* (*i* ≤ *MaxIter*) **do**  
$p\leftarrow \underset{p}{\arg\max}$ {*γ*(***p***), *q* | Constraints of Equation (3)}  **if** γ (***p***, *q*) = 0 **then**   ***p**_o_* ← *p*   *Convergence* = *true*  **else if***γ*(*p,q*)≤*ε***then**   ***p***_*ε*_ ← ***p***   *Convergence* = *true*  **else**   
$\begin{array}{ll} q\leftarrow & \frac{\sum_{k=1}^{K}C_{k}}{p_{c}+ϒ\sum_{k=1}^{K}p_{k}}\\ i\leftarrow & i+1 \end{array}$  **end if** **end while**


We convert our optimization problem given in Equation (3) to one resembling Equation (11) and then propose iterative a *ε*-optimal algorithm to get power allocation. Algorithm 1 presents the pseudo code of the proposed iterative scheme. Algorithm 1 is an iterative algorithm based on the Dinkelbach method for power allocation. Now, we will prove the convergence of the algorithm.

**Theorem 2.** *The iterative algorithm will always converge to a ε-optimal solution*.

*Proof.* Let N (**p**) = *C_k_*, *D*(**p**) = *p_c_* + Σ*_k_ p_k_* and *F*(*q*) = max{*γ*(***p**, q*)|*C*1, *C*2 of Equation (3)} = max{*N*(**p**) −*qD*(**p**)|*C*1, *C*2 of Equation (3)}. In order to prove the theorem, we need the following two Lemmas.

**Lemma 1.** ∃*q such that F*(*q*) = 0.

*Proof*: Using the *ε* − *δ* definition of continuity, we can prove that *F*(*q*) = 0 is continuous in *q*. Furthermore, $\underset{x\to \infty }{\lim}F(q)=-\infty$ and $\underset{x\to -\infty }{\lim}F(q)=+\infty$. By the intermediate value theorem (IVT), ∃*q*, such that *F*(*q*) = 0.

**Lemma 2.** *F*(*q*) *is decreasing in q.*

*Proof:* Take *q*_1_ < *q*_2_, and let p* maximize *N* (**p**) − *qD*(**p**), subject to *C*1, *C*2 of Equation (3). Then:

$$\begin{array}{ll} F\left(q_{2}\right) & =\max\left\{N\left(p\right)-q_{2}D(p\right\}\\ & =N\left(p^{*}\right)-q_{2}D\left(p^{*}\right)<N\left(p^{*}\right)-q_{1}D\left(p^{*}\right)\\ & \leq \max\left\{N\left(p\right)-q_{1}D(p)\right\}\\ & =F(q_{1}) \end{array}$$

We shall now prove the theorem. Note that it is sufficient to show that *γ*(***p***, *q*) becomes smaller than *ε* with the number of iterations. Since *F*(*q*) = max{*γ*(***p***, *q*)}, we only need to show that *F*(*q*) becomes smaller than *ε*. We now show that *q* is non-increasing in successive iterations of the algorithm. If we use the subscript, *n*, to denote the values of variables on the *n*th iteration, we have:

$$\begin{array}{ll} 0 & =N\left(p_{n-1}\right)-q_{n}D\left(p_{n-1}\right)\\ & \leq \max\left\{N\left(p_{n-1}\right)-q_{n}D\left(p_{n-1}\right)\right\}\\ & =F(q_{n})\\ & =N\left(p_{n}\right)-q_{n}D\left(p_{n}\right)\\ & =q_{n+1}D\left(p_{n}\right)-q_{n}D\left(p_{n}\right)\\ & =(q_{n+1}-q_{n})D\left(p_{n}\right) \end{array}$$

Now, it follows that *q_n_*_+1_ ≥ *q_n_*, because *D*(***p**_n_*) > 0. By Lemma 1, *F*(*q*) is decreasing in *q*, and we just proved that *q* is non-increasing in successive iterations of the algorithm. Therefore, *F*(*q*) is non-increasing in successive iterations of the algorithm. By Lemma 2, *F*(*q*) does become zero, and it follows that *F*(*q*) does become smaller than *ε.*

### 4.1. Working Principle of the Iterative Algorithm

At the start of the algorithm, it initializes *q*, *ε* and iteration counter, *i*, where *q* is the ratio between total throughput and total power. We can define the fractional objective function, Equation (3), as $\gamma \left(p,q\right)\leftarrow \sum_{k=1}^{K}C_{k}-q\left(p_{c}+ϒ\sum_{k=1}^{K}p_{k}\right)$. The algorithm iteratively solves the concave optimization problem: $\underset{p}{\arg\max}${*γ*(***p***, *q*) | *C*1 and *C*2 of Equation (3). With successive iterations of the algorithm, the value of *q* decreases. For every *q*, the power vector, *p*, that maximizes $\sum_{k=1}^{K}C_{k}-q\left(pc+ϒ\sum_{k=1}^{K}pk\right)$ is found. The algorithm terminates when *q* is zero or less than the given *ε* value. According to the Dinkelbach Theorem, at this terminal point of the algorithm, the *p* value is either optimal or *ε*-optimal. If the value of *q* is zero, then *p* is the optimal power allocation; otherwise, *p* is the *ε*-optimal power allocation.

## 5. Simulation Results

In this section, we present simulation results to demonstrate the performance and convergence of the proposed iterative scheme. The impact of network parameters (e.g., number of SSNs, number of PUs, interference threshold) is also investigated. Convex fractional programming is used to get the optimal energy efficient power allocation. Iterative algorithm is implemented for the *ε*-optimal solution.

In all simulations, the secondary users’ channel gain, *h*, is modeled as [36]:

(12) $$h=\Phi K_{o}{\left(\frac{d_{o}}{d}\right)}^{\beta },$$

where *K_o_* is a constant that depends on the antenna characteristic and average channel attenuation, *d_o_* is the reference distance for the antenna far field, *d* is the distance between the transmitter and receiver, *β* is the path loss constant and Φ is the Rayleigh random variable. Since this formula is not valid in the near field, in all the simulation results, we assume that *d* is greater than *d_o_*. In all the results, *d_o_* = 20 m, *K_o_* = 50,*β* = 3 and *N_o_* = 1*μ* W/Hz. The SSNs and PUs are uniformly distributed. The maximum coverage distance of the base station is set to 1,000 m. The total circuit power, *P_c_*, is set to 10^−6^ W. For primary user protection, we use IEEE 802.22 WRAN standard recommendations. The channel gain between the secondary and primary user is the same as mentioned in Equation (1)—*i.e.*, $g_{m,k}={\tilde{g}}_{m,k}G_{o}{\left(\frac{d_{o}}{d_{m,k}-R_{m}}\right)}^{\beta }$. Since this formula mentioned in Equation (1) is not valid in the near field, in all the simulation results, we assume that *d* – *R_m_* is always greater than the reference distance, *d_o_*. We set *d_o_* = 10 m, *G_o_* = 50, *β* = 3 and *N_o_* = 1*μ*W/Hz. Without loss of generality, we assume that *R_1_* = *R_2_* = ⋯ = *R_M_* = *R*. The PU's protected distance, *R_m_*, is set to 10 m.

**Figure 4.**
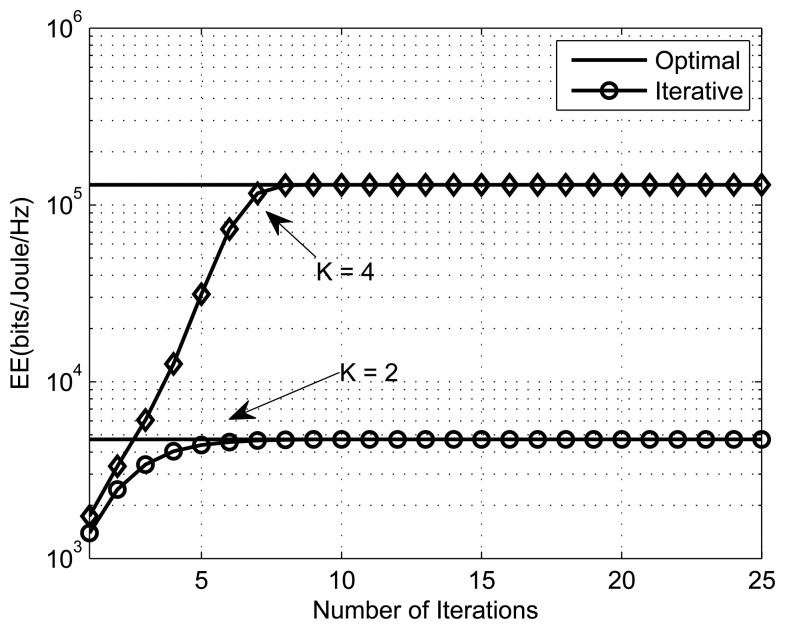
Performance of the iterative algorithm with the number of SSNs, *K* ={2,4}.

**Figure 5.**
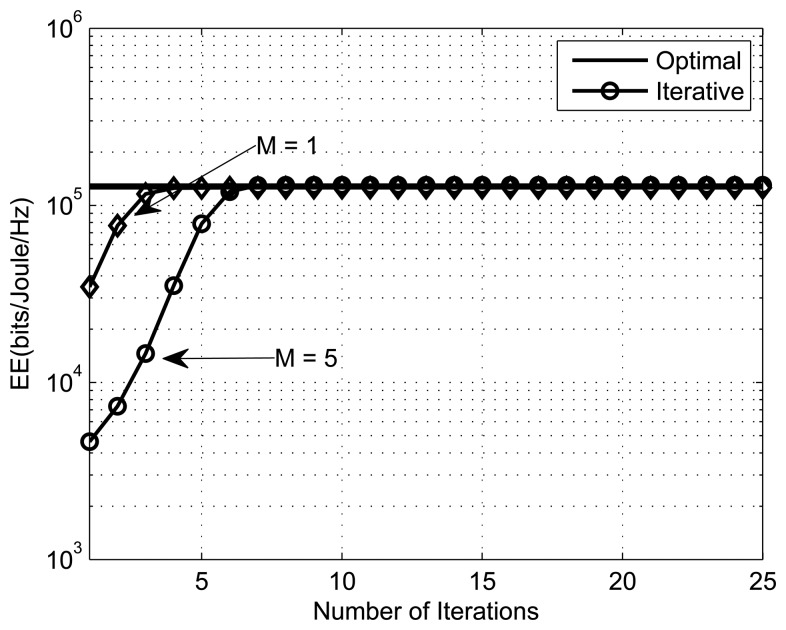
Performance of the iterative algorithm with the number of PUs, *M* = {1,5}.

**Figure 6.**
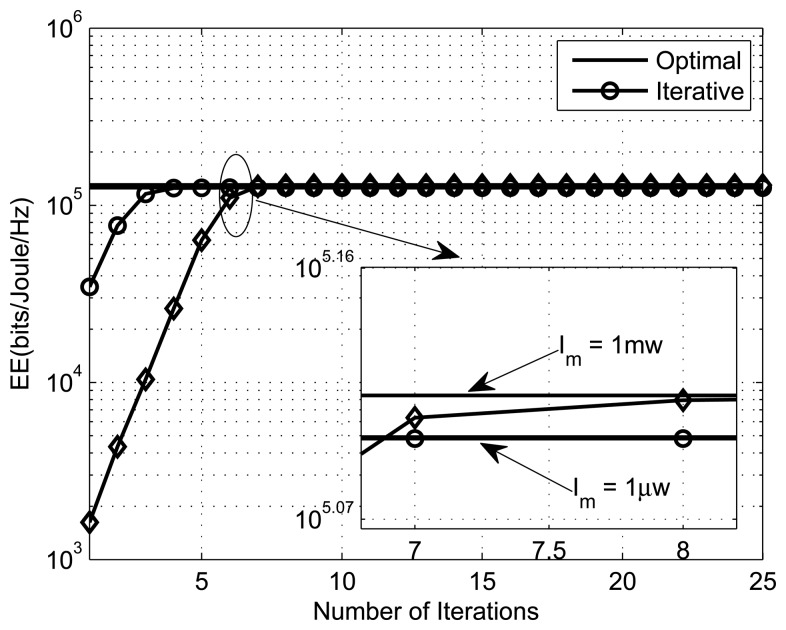
Performance of the iterative algorithm with interference thresholds, *I_m_* = {1*μ* W, 1 mW}.

The convex fractional program (CFP) is used to get the optimal energy-efficient power allocation. Figure 4, Figure 5 and Figure 6 present the energy efficiency plots with the number of iterations for different numbers of SSNs, PUs and interference thresholds, respectively. The parameters for Figure 4, Figure 5 and Figure 6 are (*K,M,I_m_,ε*,Y) = ({2, 4}, 1, 10*μW*, 10^−4^,1), (4, {1, 5}, 1*μW*, 10^−4^,1) and (4,1, {1*μW*, 1*mW*}, 10^−4^,1), respectively. From the simulation results, we can see that the iterative algorithm converges to the optimal solution within ten iterations for all different scenarios (different SSNs, PUs, *etc*). We can also observe that the EE increases with the number of secondary sensor nodes. This is due to the fact that with more SSNs, there is more freedom in power allocation. We also observe that the energy efficiency decreases with the increase in the number of primary users, because the optimization problem has a greater number of constraints to satisfy.

**Figure 7.**
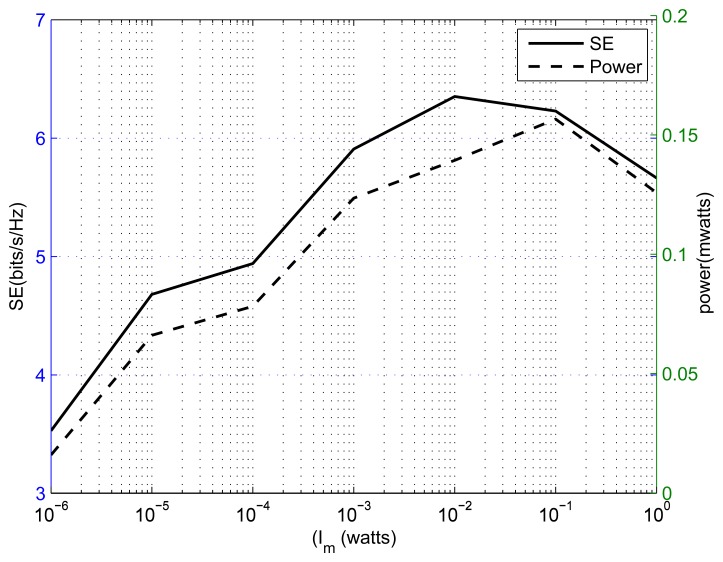
Spectral efficiency and total transmission power *vs*. interference threshold.

**Figure 8.**
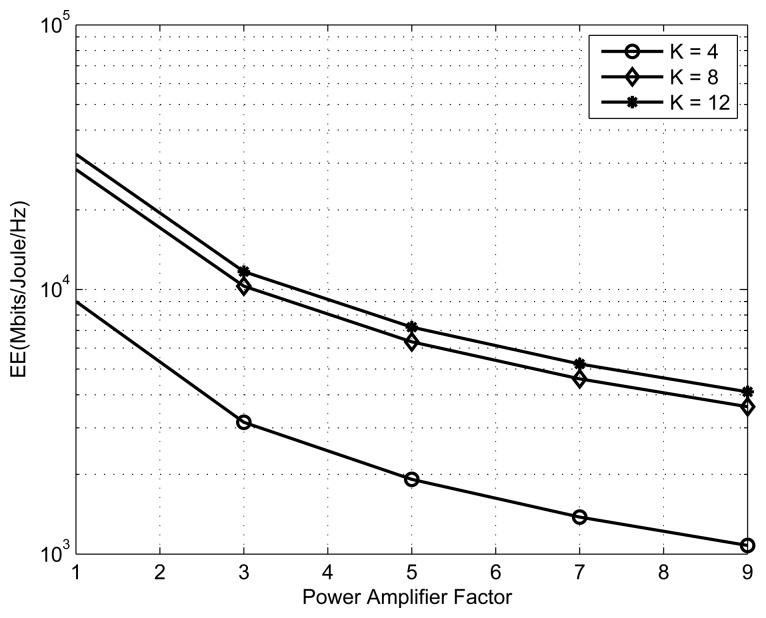
EE *vs*. PAF.

Figure 7 shows spectral efficiency and transmission power *vs*. the interference threshold plot for *K* = 8 SSNs and *M* = 1 primary user. From the result, we can observe that the increase in the interference threshold always benefits the energy efficiency, but that is not always true for spectral efficiency, because spectral efficiency is a non-decreasing function of transmitted power, while energy efficiency is not an increasing function of transmitted power. Figure 8 presents the effect of the power amplifier scaling factor on the performance of an energy-efficient cognitive radio sensor network. This figure shows energy efficiency *vs*. the power amplifier factor plot for different numbers of SSNs. The number of primary users is one and the interference threshold is set to 100*mW*. From the result, we observe that increase in the power amplifier scaling factor eventually decreases the energy efficiency. This is due to the fact that with an increase in the power amplifier scaling factor, most of the energy is wasted in the power amplifier. An optimum power amplifier is still an open area for research for energy-efficient cognitive radio sensor networks. Hopefully, in the near future, advances in electronic hardware will give us better power amplifiers.

## 6. Conclusion

In this paper, we considered the analysis of energy efficiency in a cognitive radio sensor network. The proposed energy-efficient optimization problem is not a convex optimization problem. We proposed two transformation techniques to convert the non-convex optimization problem into a convex optimization probe. We presented that by using Charnes-Cooper transformation, the problem can be transformed into a concave optimization problem. We also investigate an iterative parametric solution to the non-convex problem. The Dinkelbach method is used to solve the parametric approach. The parametric approach gives the *ε*-optimal solution. The performance of the iterative parametric method was compared with the optimal solution for different system parameters.

## Figures and Tables

**Table 1.** Notations.

**Symbol**	**Definition**
*K*	Number of secondary sensor nodes (SSNs)
*M*	Number of primary users (PUs)
*p_c_*	Static circuit power of the source in the transmit mode
*I_m_*	Interference threshold at *m*th PU
*ε*	Maximum tolerance between theoretical optimal and *ε*-optimal solution
*q*	Ratio between total throughput and total power
*β*	Path loss exponent
*R_m_*	*m*th PU-protected distance
*d_o_*	Reference distance for the antenna far field
*d*	Distance between transmitter and receiver
Φ	Rayleigh random variable
*p_k_*	Transmitted power of *k*th SSN
*C_k_*	Throughput of *k*th SSN link
*g_m,k_*	Channel gain between the *m*th PU and *k*th SSN
*h_k_*	Channel gain between the *k*th SSN and secondary base station (SBS)
Γ	Energy efficiency (EE)
*P_o_*	Optimal power vector
*P_ε_*	*ε*-optimal power vector
Y	Power amplifier factor

## A. Proof of Theorem 1

We can write the objective function in Equation (6) as $t\sum_{k=1}^{K}\log\left(1+\frac{y_{k}h_{k}}{tN_{0}}\right)=\left(\sum_{k=1}^{K}\log\left(t+y_{k}\xi_{k}\right)\right)-Kt\log t$. The Lagrangian function of Equation (6) is given by:

(13) $$L\left(y,t,\phi ,\lambda ,\upsilon ,\mu \right)=\left(t\sum_{k=1}^{K}\log\left(t+y_{k}\xi_{k}\right)\right)-Kt\log t-\sum_{m-1}^{M}\phi_{m}\left(\sum_{k=1}^{K}y_{k}g_{m,k}-tI_{m}\right)-\upsilon \left(t_{pc}-1+ϒ\sum_{k=1}^{K}y_{k}\right)+\sum_{k=1}^{K}\lambda_{k}y_{k}+\mu t,$$

where *φ*, ***λ***, *υ*, *μ* are Lagrangian coefficients. Let $y_{k}^{*}$ and *t** be the optimal solutions. The KKT conditions can be written as:

(14) $$\sum_{m=1}^{M}\phi_{m}\left(\sum_{k=1}^{K}y_{k}^{*}g_{m,k}-tI_{m}\right)=0$$

(15) $$\left(tp_{c}-1+ϒ\sum_{k=1}^{K}y_{k}^{*}\right)=0$$

(16) $$\lambda_{k}y_{k}^{*}=0\forall k$$

(17) μt=0

(18) $$\begin{array}{r} \lambda_{k}\geq 0,\forall k\\ \phi_{m}\geq 0,\forall m\\ \mu \geq 0 \end{array}$$

(19) $$\frac{1}{\ln2}\frac{t^{*}\xi_{k}}{t^{*}+y_{k}^{*}\xi_{k}}+\lambda_{k}-\sum_{m=1}^{M}\phi_{m}g_{m,k}-\upsilon =0,\forall k$$

(20) $$\frac{\partial L}{\partial t^{*}}=0$$

We can easily eliminate *λ_k_* and *μ* from the above KKT conditions. The simplified KKT equations are:

(21) $$\sum_{m=1}^{M}\phi_{m}\left(\sum_{k=1}^{K}y_{k}^{*}g_{m,k}-tI_{m}\right)=0$$

(22) $$\left(tp_{c}-1+ϒ\sum_{k=1}^{K}y_{k}^{*}\right)=0$$

(23) $$\phi_{m}\geq 0,\forall_{m}$$

(24) $$\frac{1}{\ln2}\frac{t^{*}\xi_{k}}{t^{*}+y_{k}^{*}\xi_{k}}-\sum_{m=1}^{M}\phi_{m}g_{m,k}-\upsilon =0,\forall k$$

From (24) and constraints, $y_{k}^{*}\geq 0$ and *t** > **0**, we get:

(25) $$\left(\frac{y_{k}^{*}}{t^{*}}\right)={\left(\frac{1}{\ln2\left(\upsilon +\sum_{m=1}^{M}\phi_{m}g_{m,k}\right)}-\frac{1}{\xi_{k}}\right)}^{+},\forall k.$$

From the CCT, we have $y_{k}^{*}=p_{k}^{*}t^{*}$. Hence, from (25), we get the optimal power allocation as:

(26) $$p_{k}^{*}=\left(\frac{y_{k}^{*}}{t^{*}}\right)={\left(\frac{1}{\mathrm{In}2\left(\upsilon +\sum_{m=1}^{M}\phi_{m}g_{m,k}\right)}-\frac{1}{\xi_{k}}\right)}^{+},\forall k.$$
